# Pediatric early warning score and unplanned readmission with influenza at emergency observation room

**DOI:** 10.3389/fped.2025.1674746

**Published:** 2026-01-16

**Authors:** Wanting Xu, Anji Liu, Wen Xu, Qinxin Zheng, Lei Li, Yan Liu, Xiaoping Lei, Lan Kang, Wenbin Dong

**Affiliations:** 1 Department of Neonatology, Children’s Medical Center, The Affiliated Hospital of Southwest Medical University, Luzhou, Sichuan, China; 2 Department of Perinatology, The Affiliated Hospital of Southwest Medical University, Luzhou, Sichuan, China; 3 Pediatrics, Sichuan Clinical Research Center for Birth Defects, Luzhou, Sichuan, China; 4 Pediatrics, West China School of Medicine, Sichuan University, Sichuan University affiliated Chengdu Second People's Hospital, Chengdu Second People's Hospital, Chengdu, Sichuan, China

**Keywords:** children, emergency observation room, influenza, pediatric early warning score, unplanned readmission

## Abstract

**Background:**

This study explored the predictive value of the pediatric early warning scores (PEWS) in recognizing clinical deterioration and identifying children with influenza with unplanned readmission to the emergency observation room (EOR) within 72 h.

**Methods:**

A total of 196 children were included in this single-center case–control study conducted between 1 January and 30 June 2024. PEWS scores and receiver operating characteristic curves were used to evaluate the correlations between the scores and the likelihood of influenza necessitating readmission.

**Results:**

For each 1-point increase in PEWS score, the risk of unplanned readmission significantly increased with unadjusted PEWS of [OR 9.98 (1.54–64.73); *P* = 0.016] and adjusted PEWS of [OR 10.53 (3.47–31.98); *P* < 0.001], respectively. The time to reach the highest PEWS was significantly longer in patients who experienced unplanned readmissions (*P* < 0.01). Among the non-readmitted patients, 100 out of 147 (68.03%) took oseltamivir orally, compared with only 10 out of 49 (20.41%) patients with unplanned readmissions within 72 h (*P* < 0.001). A PEWS cutoff score of 3 indicated a relatively high sensitivity of 61.2% and specificity of 96.6%, with a positive likelihood ratio (PLR) of 18.0 and a negative likelihood ratio of 0.4.

**Conclusions:**

PEWS is potential for predicting unplanned readmissions with similar symptoms in children with influenza, demonstrating it may be an essential tool for enhancing patient care and outcomes.

## 1 Introduction

Influenza virus (IV) is classified under the *Orthomyxoviridae* family of single-stranded RNA viruses (1). The Chinese National Influenza Center reported that influenza is a significant public health concern in China, especially for children. Between 2004 and 2020, the average annual incidence of influenza in mainland China was 31.57 cases per 100,000 people. In 2009 and 2019, there were notable outbreaks that exceeded the expected values by 142% and 323%, respectively (2). Influenza A (H3N2) was the most common subtype during the 2023/2024 season, and influenza A (H1N1) pdm09 emerged as the most common circulating strain during the 2024/2025 season (3). Notably, the burden of influenza was more pronounced in young children aged 0–4 years than in the 2020–2021 season, emphasizing the potential severity of seasonal influenza in this demographic (4, 5). Severe influenza is identified when at least one of the following clinical manifestations is present: dyspnea, tachypnea, or hypoxia; radiological signs of lower respiratory tract disease; central nervous system involvement, including convulsions, encephalopathy, or encephalitis; severe dehydration; acute renal failure; septic shock; and exacerbation of underlying chronic diseases (6). Owing to the prevalent resistance of epidemic influenza virus strains to existing antiviral strategies, children with severe influenza are at increased risk of clinical deterioration (7). In children, the respiratory system is not completely developed, with narrow respiratory tracts, less alveolar surface available for oxygen exchange, and less ability to regulate breathing, making them prone to airway obstruction and acute respiratory failure (8, 9). Disease progression and outcomes are often predictable, and in children, clinical deterioration typically precedes physical signs by approximately 24 h (10, 11).

The pediatric early warning scoring (PEWS) system was initially used to assess clinical deterioration among pediatric patients, focusing on three fundamental vital signs (12). The PEWS system was then further developed and standardized in clinical practice, according to a 2023 news item describing its rollout throughout England (13). PEWS is an objective evaluation tool that, when combined with clinical action, can improve the recognition and treatment of children to prevent clinical deterioration (14–16). Failure to recognize signs and clinical decompensation, lack of knowledge, and failure to seek timely advice are major reasons for urgent ICU transfer and poor clinical outcomes (17, 18). Age-specific PEWS systems are essential for pediatric inpatients, considering the physiological variations in heart rate, respiratory rate, and blood pressure from neonatal to adolescent stages. In the emergency room, pediatric patients experience unpredictable disease transitions that can occur at any time (19). Although evaluations of individual physicians are usually appropriate, there is a trend toward more objective indicators of the need for transfer to the PICU, such as elevations in PEWS scores (20, 21). PEWS serves as a standardized, real-time, and visible tool for the early identification of clinical decompensation, effectively recognizing clinical deterioration without age limitations (22).

This study investigated whether PEWS may also be potential in identifying children infected with influenza who are under consecutive 24 h observation in the pediatric emergency observation room (EOR) and are at a high risk of early (within 72 h) emergency readmission for the same symptom. Therefore, it is hypothesized that elevated PEWS scores, both upon discharge from the EOR and within the first 24 h, may predict an unplanned readmission within 72 h due to acute clinical deterioration.

## 2 Materials and methods

### 2.1 Study design and data collections

**Figure 1:**
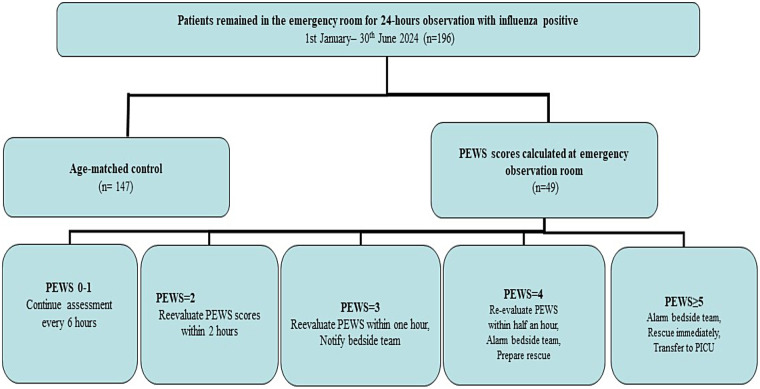
Flowchart of cases in the research. PEWS, pediatric early warning scores.

A single-center case–control clinical study was conducted to examine the correlation between the highest PEWS score during the 24 h observation and the PEWS score upon discharge in the pediatric EOR (Figure 1). The scoring system comprised three vital elements, namely, respiratory, cardiovascular, and behavioral signs of critical deterioration events (CDEs), with each item scored on a scale from 0 to 3 (Supplementary Material S1). Two additional points were assigned for continuous administration of inhalation medication. The cumulative scores ranged from 0 to 11, with zero indicating a healthy physiological state and higher scores indicating an increased risk of clinical deterioration. Respiratory and heart rates were assessed in relation to typical vital signs across the various age groups (Supplementary Material S2). This was a retrospective, single-center, observational case–control study based on routinely collected clinical records in the pediatric EOR. According to local regulations for non-interventional retrospective studies, the study was not registered in a clinical trial registry because no experimental interventions beyond standard care were introduced.

### 2.2 Settings

An EOR is a short-stay unit belonging to the emergency department staffed by a 24 h shift resident doctor who decides whether pediatric patients should continue under observation, notify the bedside team, or be sent home. All patients were observed in this unit for 24 h and were accompanied by at least one guardian. The research leader modified the tool based on the literature, and an updated version was presented to the bedside team to assess its practicality and validity. The revised manuscript has been reviewed and approved by the same team.

### 2.3 Data sources

A total of 196 children were diagnosed with influenza and treated at a tertiary hospital between 1 January and 30 June 2024. The inclusion criteria were as follows: (1) children aged from 2 to 14 years old; (2) influenza antigen-positive on nasal swab and real-time fluorescence quantitative polymerase chain reaction (RT-qPCR) test; (3) first influenza diagnosis within 48 h and willingness to undergo medical observation for 24 h in the EOR; (4) clinical signs of influenza, including high fever, cough, and elevated breathing and heart rates; and (5) comprehensive clinical data anticipated. The exclusion criteria were as follows: (1) children with genetic metabolic diseases, a history of asthma, bronchopulmonary dysplasia, or psychiatric disorders; (2) those who were discharged home, admitted to other units, or returned to the emergency department; and (3) those who were readmitted with different symptoms from the first presentation. At the EOR visit, venous blood samples were obtained as part of routine clinical care. Procalcitonin (PCT) was measured using an automated chemiluminescent immunoassay, and alanine aminotransferase (ALT) and creatine kinase (CK) were tested by standard enzymatic methods on an automated biochemical analyzer. Abnormal elevations were defined according to the hospital laboratory reference ranges, for example, PCT > 0.5 ng/mL, ALT > 40 U/L, and CK > 200 U/L.

### 2.4 Operation strategy

In our pediatric EOR, PEWS scoring was implemented as a standardized clinical tool before data collection for this retrospective study. Six resident pediatricians working in the emergency department participated in a structured 1-day training course on the use of the locally adapted PEWS system, led by the research leader. This training included (1) detailed instruction on the PEWS chart and age-specific ranges for heart rate and respiratory rate; (2) clarification of operational definitions for each respiratory, cardiovascular, and behavioral item; and (3) case-based simulations where residents discussed disagreements to come to a consensus and scored standardized clinical scenarios. All scores were documented in an online Microsoft Excel software, in which PEWS items were pre-specified to minimize subjectivity and transcription mistakes. The scores were recorded in electronic health records. PEWS was automatically classified into low score (0–2), median score (3–4), and high score (≥5) groups with distinct color codes. An attending pediatrician on duty reviewed PEWS charts with medium or high scores during the first implementation period, and any discrepancies were discussed to support consistent scoring practice. RT-qPCR and a positive rapid antigen test were conducted for the diagnosis of influenza at the index visit, and the results were entered into Microsoft Excel online. Repeat testing was not required for readmissions within 72 h as a previous positive test was recorded without a different diagnosis. Following the provision of care, two research investigators independently used Microsoft Excel online software to confirm eligibility, for example, inclusion, exclusion, and “similar symptom” definition. This verification was not blinded; rather, it was a double check of the documentation based on the earlier standards. In addition, PEWS score 0–1: No treatment is required, but assessment should be performed every 6 h. PEWS score 2: Reevaluate clinical symptoms such as high fever, elevated breathing, and heart rate; calculate fluid balance; and reevaluate PEWS scores within 2 h. PEWS score 3: Examine clinical symptoms, reassess PEWS scores within an hour, and inform the bedside team. PEWS score 4: Reevaluate PEWS within half an hour, alarm the bedside team, organize rescue, and prepare for transfer to the PICU. PEWS score > 5: Alert the bedside team, notify the general inpatient and PICU doctors to arrive at the scene within 5 min, immediately rescue, and transfer to the PICU.

### 2.5 Eligibility criteria

**Figure 2:**
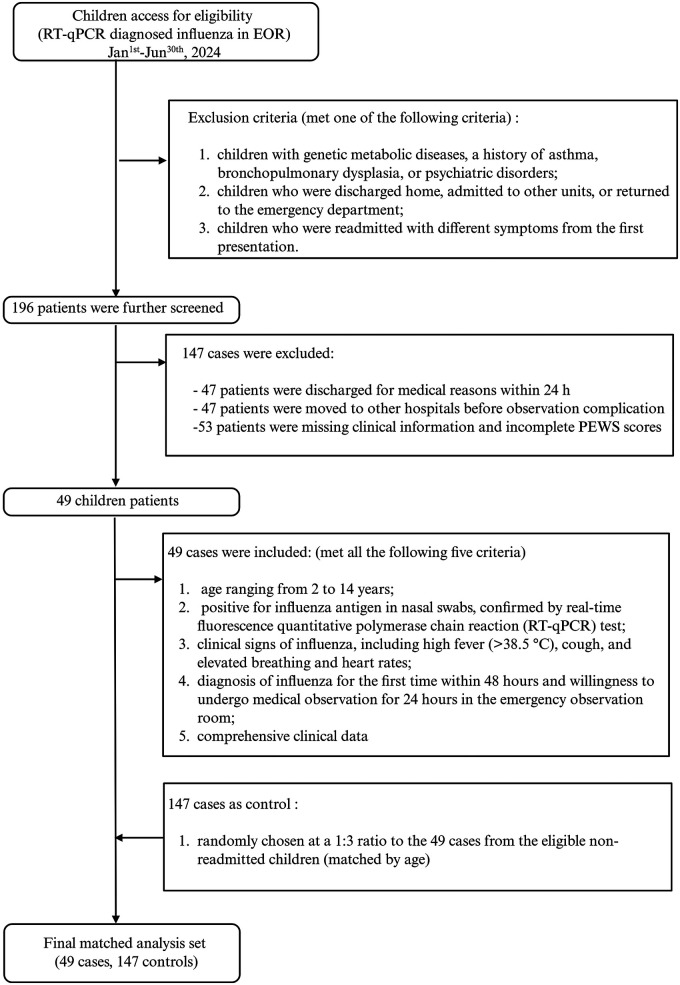
Flow diagram details of the patient screening process. The 147 case controls are totally distinct from the 147 excluded patients. EOR, emergency observation room.

Specific and objective criteria were used to assess the time required to achieve clinical stability after the initiation of continuous observation for influenza in the pediatric emergency department. Mechanical ventilation, vasoactive drugs such as dobutamine or epinephrine, and cardiopulmonary resuscitation are necessary for clinical deterioration (23, 24). The potential control pool was made up of all children with laboratory-confirmed influenza during the study period who satisfied the inclusion and exclusion criteria and were not readmitted to the EOR within 72 h. For the 72 h unplanned readmission endpoint, “similar symptoms” were defined *a priori* as recurrence of the index chief-complaint constellation, for example, fever with cough, dyspnea, or wheezing, along with age-adjusted tachypnea or tachycardia as operationalized by the study PEWS items, without the emergence of a new focal chief complaint that indicated a different syndrome. Returns with inconsistent symptom profiles were not involved. The highest PEWS score was determined after continuous monitoring for 24 h and a review of medical charts. Resident pedestrians recorded their final PEWS scores before the child was released from the EOR. At a 1:3 case–control ratio, stratified by age, controls were chosen at random from the pool of children who satisfied all inclusion criteria, had complete clinical data, and were not readmitted to EOR within 72 h using a computer-generated sequence. The resulting age-stratified risk sets were then analyzed using conditional logistic regression to account for the case–control structure. Returns where the main reason for readmission is a new focal diagnosis, for example, radiologically confirmed pneumonia not present initially, or bacterial co-infection with a different clinical symptom, were excluded according to the protocol to ensure the endpoint reflected worsening of the initial influenza illness. Children who tested positive for influenza were identified as age-matched controls and did not need to be readmitted to the observation room after discharge from the EOR (Figure 2).

### 2.6 Evaluation indicators

PEWS scores during the observation period and when leaving the observation room were recorded. The association between PEWS scores and the risk of unplanned readmission was investigated using conditional logistic regression analysis adjusted for the use of continuous inhalation medications, and odds ratios (ORs) and their 95% CIs were calculated. Receiver operating characteristic (ROC) curves were used to evaluate the predictive performance of both the highest and final PEWS scores recorded in the EOR as well as the adjusted versions of these scores.

Three age groups were evaluated to compare the time and temperature associated with the highest PEWS scores: 2–3 years, 4–6 years, and 7–14 years. The comparison also considered patients who were not readmitted and those who had an unplanned readmission to an emergency observation unit within 72 h.

The medical records of the children were used to gather clinical variables and demographic data. Upon admission, the following information was gathered: demographic information, influenza virus type, presence of complications, fever duration, time to achieve an antipyretic effect following medication, relevant laboratory markers, and influenza vaccinations. PCT, ALT, and CK were predetermined as the laboratory reference indicators reflecting the severity of the disease, as elevated PCT levels may indicate the presence of bacterial co-infection. Elevated ALT and CK levels may reflect liver function damage and the existence of viral myositis in severe influenza. Therefore, these markers were collected as potential supportive predictors of clinical deterioration alongside PEWS. Descriptive statistics were used to evaluate the pattern of demographic characteristics and PEWS scores among children readmitted to the pediatric emergency department for the same symptoms within 72 h.

Analysis of the relationship between the highest PEWS recorded in the observation room and the final PEWS scores upon discharge included adjustments to account for the use of continuous inhalation medications. The cutoff scores for unplanned readmission within 72 h for children infected with influenza were determined, and the PEWS scores in readmitted patients were adjusted for analysis. The clinical utility of the predictive model in each group was assessed by determining its sensitivity and specificity. In addition, the likelihood ratios for positive and negative outcomes were analyzed and compared with those of studies that used populations without readmission.

### 2.7 Statistical analyses

The Wilcoxon rank-sum test was used to assess the association between PEWS and the occurrence of unplanned readmissions because of the non-normal distribution of variables. Spearman's correlation coefficient was applied to measure the correlation between the final PEWS recorded upon discharge and the highest PEWS observed in the pediatric EOR during the initial 24 h period. Finally, the Wilcoxon signed-rank test was performed to examine the difference between the median PEWS scores at discharge and those recorded during the peak 24 h observation period. The non-parametric DeLong test was used to compare the area under the receiver operating characteristic curves (AUCs) for correlated ROC curves, and 95% confidence intervals for AUCs were calculated. Statistical analyses were performed using SPSS version 22.0 and GraphPad Prism version 9.0. A significance level of *P* < 0.05 was applied for all tests.

## 3 Results

**Figure 3:**
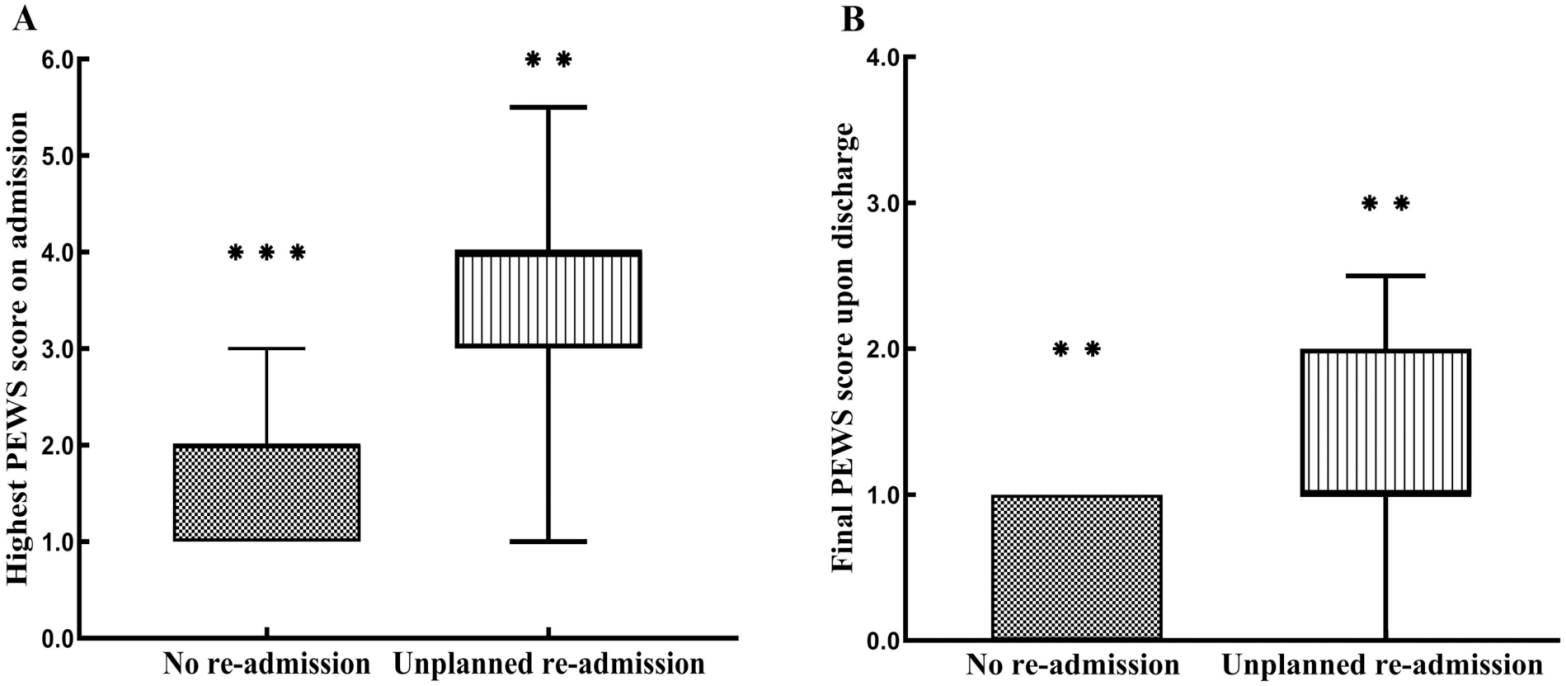
PEWS scores at the emergency observation room. **(A)** The highest PEWS score was for the 147 children who did not require readmission at the emergency observation room within 72 h, compared with 49 children unplanned readmission at the emergency observation room within 72 h. **(B)** Final PEWS score upon discharge for the 147 children who did not require readmission within 72 h, compared with 49 children unplanned readmission within 72 h. The median values were 0 (with an interquartile range of 0–1) for the group without the condition and 1 (with an interquartile range of 1–2) for the group with the condition. The difference between these medians was statistically significant (*P* < 0.001), as determined by the Wilcoxon rank-sum test. The box indicated the median with standard deviation (top of box = 95th percentile, bottom of the box = 5th percentile). The top whisker represents 95% percentage of the upper PEWS scores. The bottom whisker represents 5% percentage of the bottom PEWS scores. Outliers were indicated as dots above the whisker. *vs. no-readmission group. **P < 0.01, ***P < 0.001. Two-way ANOVA analysis.

**Figure 4:**
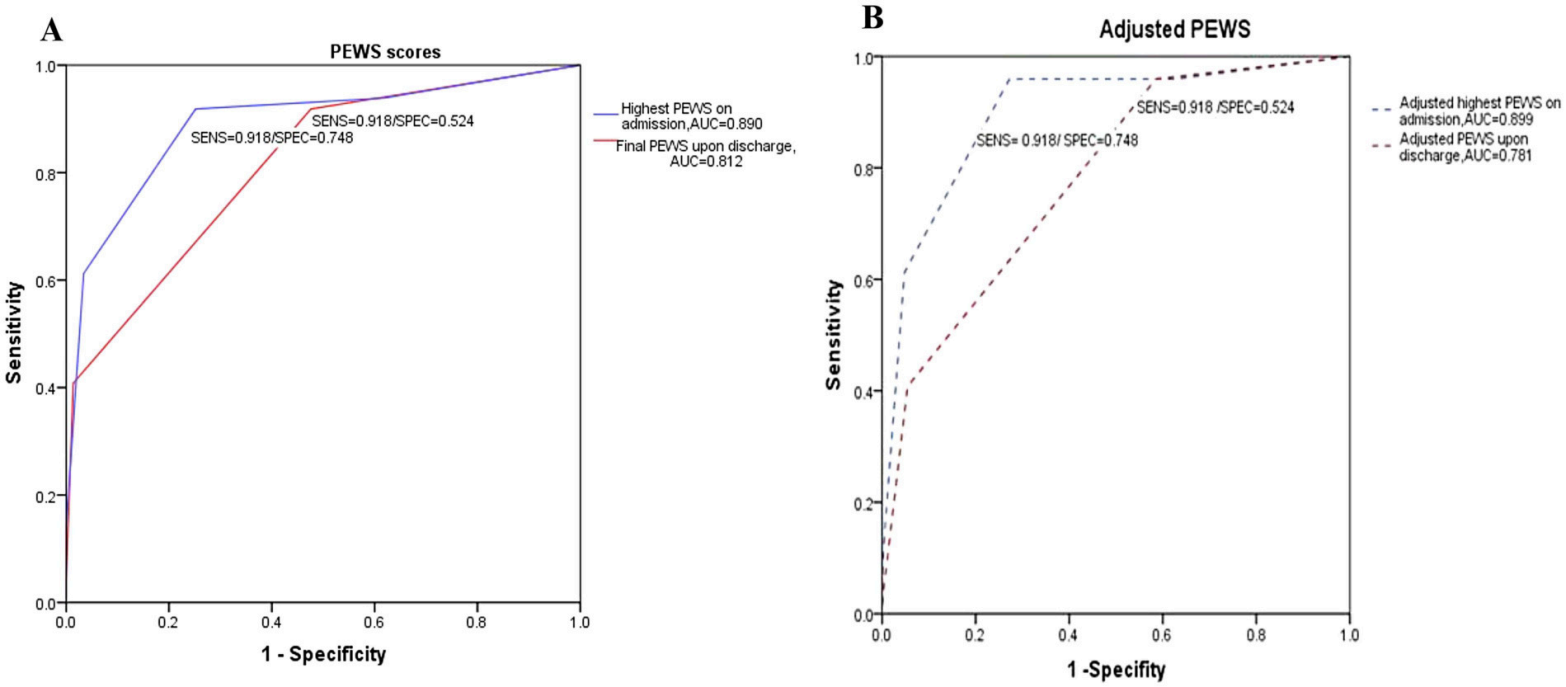
Receiver operating characteristic curves for patients infected with influenza recorded by PEWS. **(A)** Highest PEWS score on the first 24 h at the emergency observation room, AUC = 0.890, 95% CI (0.829−0.950). Final PEWS score upon discharge, AUC = 0.812, 95% CI (0.742−0.883). **(B)** Adjusted highest PEWS score on admission, AUC = 0.899, 95% CI (0.845−0.952). Adjusted PEWS score upon discharge, AUC = 0.781, 95% CI (0.709−0.853).

Three age-matched controls from the same time window among children meeting the inclusion criteria were randomly selected with full data and were not readmitted to EOR within 72 h, resulting in 147 controls. The planned 24 h observation was not completed by 47 patients. Prior to an observation complication, 47 patients were moved to other hospitals. Due to missing critical clinical data and incomplete PEWS scores, 53 patients were eliminated. Thus, 49 cases and 147 controls, in a total of 196 observations, made up the final matched analysis set. The highest PEWS within the first 24 h of constant observation was lower in the non-readmitted group than in the unplanned readmitted within 72 h group [2(1,2) vs. 4(3,4), *P* < 0.001] (Figure 3A). The final PEWS was lower in the non-readmitted group than in the unplanned readmitted within the 72 h group [0(0,1) vs. 1 (1,2), *P* < 0.001] (Figure 3B). Moreover, the predictive accuracy of the highest PEWS score observed in the EOR increased, compared with the final PEWS score upon discharge when continuous inhalation medications were added to the multivariate analysis model, with an AUC of 0.890 vs. 0.812, *P* < 0.001 (Figure 4A). PEWS score screening did not improve after adjusting continuous inhalation medication, with an AUC of the highest PEWS on admission of 0.812 vs. upon discharge of 0.781, *P* = 0.028 (Figure 4B).

**Figure 5:**
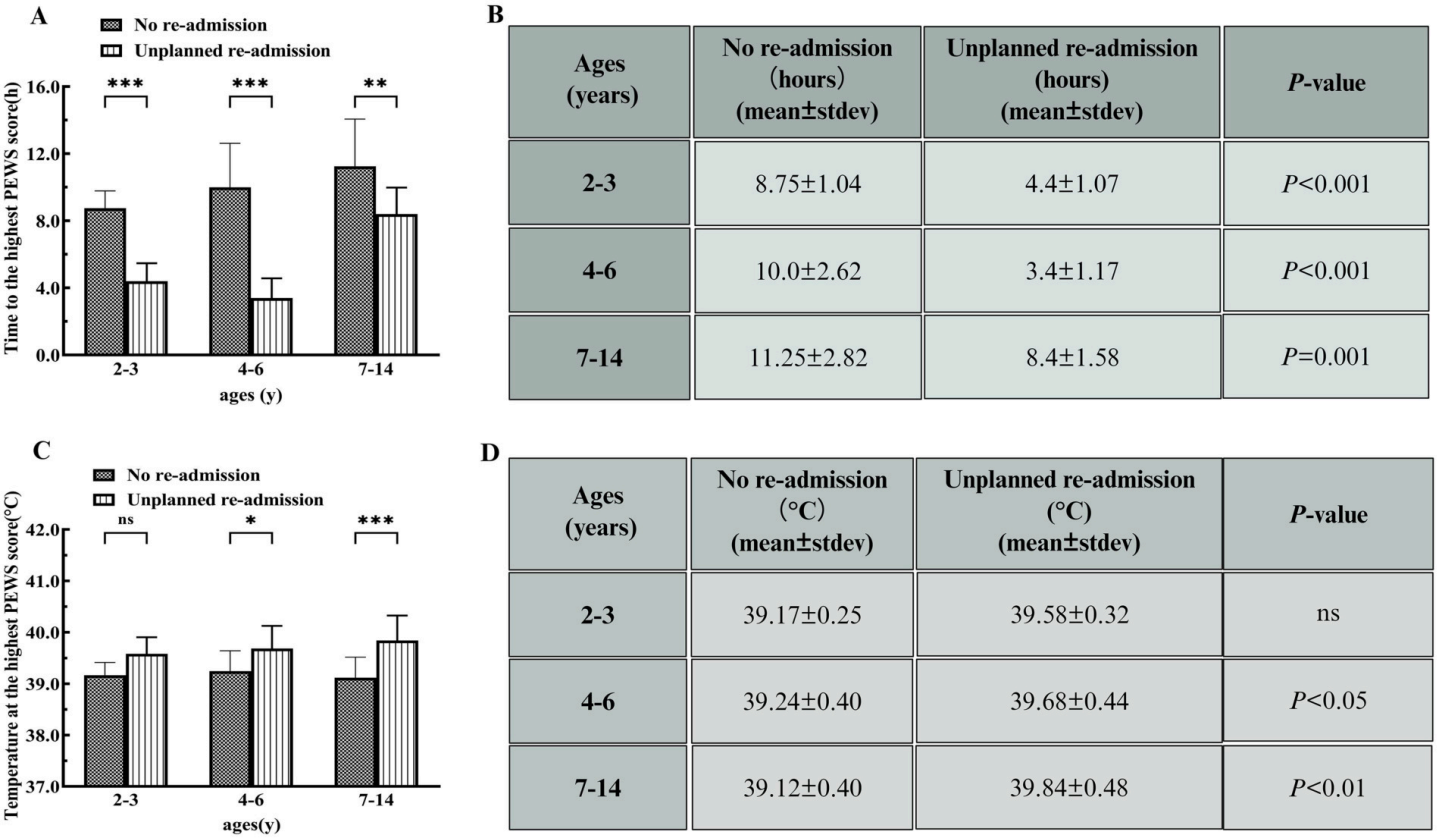
Time and temperature to the highest PEWS scores. **(A,B)** Time to the highest PEWS scores for children who were not readmitted, compared with those with an unplanned readmission. **(C,D)** Body temperature for children who were not readmitted compared with those with an unplanned readmission. Data are shown as mean ± SD. *vs. no-readmission group. *P < 0.05, **P < 0.01, ***P < 0.001, ns = no difference. Two-way ANOVA analysis.

Children aged 2–3 years with no readmission were found to have a time to the highest PEWS of 8.75 ± 1.04 h, significantly longer than the 4.4 ± 1.07 h observed in children with an unplanned readmission (*P* < 0.001). Similarly, children aged 4–6 years not readmitted had a time to the highest PEWS of 10.0 ± 2.62 h, compared with 3.4 ± 1.17 h for those readmitted (*P* < 0.001). For children aged 7–14 years, time to the highest PEWS for non-readmitted children was 11.25 ± 2.82 h, which was significantly longer than the 8.4 ± 1.58 h for those with unplanned readmission, *P* = 0.005 (Figures 5A, B). The average temperature for children ages 2–3 years without readmission was 39.16 ± 0.25 °C, with no significant difference from the 39.58 ± 0.32 °C observed in children with an unplanned readmission (*P* > 0.05). In addition, the average temperature for children aged 4–6 years who were not readmitted was 39.24 ± 0.40 °C, which was significantly lower than the 39.68 ± 0.44 °C for those who had an unplanned readmission (*P* < 0.05). The average temperature for children aged 7–14 was 39.12 ± 0.40 °C, which was considerably lower than the 39.84 ± 0.48 °C for those who had unplanned readmission, *P* < 0.001 (Figures 5C,D).

The cases and controls exhibited similar sex, median age, and white blood cell (WBC) counts for laboratory markers (Table 1). Compared with controls, case patients had a higher chance of being infected with H1N1 and H3N2 influenza viruses (77.5% vs. 61.9%, *P* = 0.047). Compared with controls, they were more likely to experience complications such as dyspnea, abnormal elevation of cardiac enzymes, prolonged high fever for >24 h, and hyperpyretic convulsions (55.0% vs. 5.3%, *P* < 0.001). The case patients exhibited a longer duration of recurrent fever that lasted longer than 24 h and a longer time to decrease fever after taking antipyretic medication than the control group (*P* < 0.001). Levels of laboratory markers, such as PCT, ALT, and CK were significantly higher in patients than in controls (*P* < 0.05). Of the 100 children who were not readmitted, 68.0% were taking oral oseltamivir. Only 10 children (20.4%) with unplanned readmissions within 72 h had taken oseltamivir orally. Statistically significant differences were observed in these proportions (*P* < 0.001). A total of 127 non-readmitted children received standard pediatric vaccinations (Table 1), accounting for 86.4% of the group. Additionally, 26 of these children were vaccinated against influenza, accounting for 17.7% of the population. A total of 43 of the children who had an unplanned readmission within 72 h had received standard pediatric vaccinations, making up 87.7% of those individuals, while only 6 had been vaccinated against influenza, accounting for 12.2%. Vaccination rates were not significantly different between the two groups.

**Table 1:** Clinical characteristics of children with influenza of unplanned readmission compared with no readmission.

Items	No readmission	Unplanned readmission	*P*-value
Number	147	49	
Gender (male)	90 (61.2%)	30 (61.3%)	>0.05
Ages(years)			>0.05
2–3 years	33 (22.4%)	11 (22.4%)	
4–6 years	42 (28.6%)	14 (28.5%)	
7–14 years	72 (49.0%)	24 (48.9%)	
Influenza virus type			0.047
H1N1	45 (30.6%)	20 (40.8%)	
H3N2	46 (31.3%)	18 (36.7%)	
BV	29 (19.7%)	7 (14.3%)	
BY	27 (18.4%)	4 (8.2%)	
Complications presented			<0.001
Dyspnea	3 (2%)	15 (30.6%)	
Abnormal elevation of cardiac enzymes	2 (1.3%)	3 (6.1%)	
Prolonged high fever over 24 h	1 (0.7%)	3 (6.1%)	
Hyperpyretic convulsion	2 (1.3%)	6 (12.2%)	
Period of continuous fever (hours)	24.42 (15.0)	43.95 (14.0)	<0.001
Antipyretic time after medication (hours)	25.6 (6.0)	35.3 (7.0)	<0.001
Laboratory marker			
White blood cell count (10^−9^/L)	7.786 (3.76)	8.921 (5.81)	0.305
Procalcitonin	0.369 (0.12)	0.494 (0.36)	0.039
Alanine aminotransferase	25.4 (14.0)	52.0 (65.0)	0.001
Creatine kinase	29.9 (15.0)	53.3 (49.0)	<0.001
Oseltamivir orally taken			<0.001
Yes	100 (68.0%)	10 (20.4%)	
No	47 (32.0%)	39 (79.6%)	
General pediatric Vaccinations acquired			0.807
Yes	127 (86.4%)	43 (87.8%)	
No	20 (13.6%)	6 (12.2%)	
Influenza vaccine acquired within a year			0.372
Yes	26 (17.7%)	6 (12.2%)	
No	121 (82.3%)	43 (87.8%)	

A significant relationship between the highest PEWS recorded within 24 h and the final PEWS at the time of discharge remained even when continuous inhalation medications were adjusted (Table 2). Univariate analysis revealed that a higher PEWS score observed during the initial 24 h period and at discharge was significantly associated with an increased risk of unplanned readmission within 72 h [OR (95% CI): 9.973 (2.82–35.24), *P* < 0.001, and 3.678 (1.22–11.10), *P* = 0.021, respectively]. After adjusting for the continuous inhalation medications, the highest PEWS scores remained a significant predictor of unplanned readmission [adjusted OR (95% CI): 10.53 (3.47–31.98); *P* < 0.001]. The final PEWS score at discharge also remained significant in the adjusted model [adjusted OR (95% CI): 9.98 (1.54–64.73); *P* = 0.016]. In the multivariate model adjusted for the use of continuous inhalation medications and oral oseltamivir, both the highest and final PEWS scores remained significant independent predictors of unplanned readmission [adjusted OR (95% CI): 5.01 (1.97–12.75), *P* = 0.001, and 0.16 (0.07–0.36), *P* < 0.001]. Furthermore, univariate analysis recognized several other factors significantly associated with an increased risk, including elevated levels of laboratory markers PCT [adjusted OR (95% CI): 3.41 (1.52–7.64); *P* = 0.003], ALT [adjusted OR (95% CI): 3.05 (1.31–7.10); *P* = 0.01], and CK [adjusted OR (95% CI): 4.72 (2.04–10.92); *P* < 0.001].

**Table 2:** Unadjusted, adjusted odds ratios and 95% confidence intervals from multivariate conditional logistic regression analysis for unplanned readmission within 72 h.

Items	Unadjusted			Adjusted^a^		
	OR (95% CI)	AUC	*P-*value	OR (95% CI)	AUC	*P-*value
PEWS scores						
Final PEWS score upon leaving	3.678 (1.22–11.10)	0.812	0.021	9.98 (1.54–64.73)	0.781	0.016
Highest PEWS score at observation	9.973 (2.82–35.24)	0.890	<0.001	10.53 (3.47–31.98)	0.899	<0.001
Clinical factors						
Continuous inhalation medication use	6.92 (2.89–16.55)	N/A	<0.001	5.01 (1.97–12.75)	N/A	0.001
Oral oseltamivir use	0.13 (0.06–0.28)	N/A	<0.001	0.16 (0.07–0.36)	N/A	<0.001
Laboratory markers						
PCT > 0.5 ng/mL	4.55 (2.14–9.66)	N/A	<0.001	3.41 (1.52–7.64)	N/A	0.003
ALT > 40 U/L	3.89 (1.76–8.61)	N/A	0.001	3.05 (1.31–7.10)	N/A	0.01
CK > 200 U/L	5.88 (2.67–12.96)	N/A	<0.001	4.72 (2.04–10.92)	N/A	<0.001

OR, odds ratio; CI, confidence interval; AUC, area under the receiver operating characteristic curve; N/A, not applicable, as an AUC was not calculated for this individual factor.

a Adjusted for continuous inhalation medications. All variables listed in the table adjusted the multivariate model.

When discharged, the PEWS scores were cutoff at 1, with a sensitivity of 91.8% and a specificity of 52.4%. However, the PLR was 1.9, and the NLR was 0.2. The most sensitive cutoff for influenza-affected patients who had the highest PEWS score during the first 24 h observation was 3, with a specificity of 96.6% and a sensitivity of 61.2%. The PLR for this cutoff was 18.0, and the NLR was 0.4%. The adjusted PEWS cutoff had an optimal combination with a sensitivity of 95.9% and specificity of 72.8%. This cutoff demonstrated a PLR of 3.5 and an NLR of 0.1 (Table 3). Clinical deterioration was observed in 20% and 14% of the patients with PEWS scores >3 and >4, respectively. The optimal cutoff value for the highest PEWS score during the initial 24 h observation was determined *post hoc* via ROC analysis. The threshold that best balanced sensitivity and specificity for predicting unplanned readmission within 72 h was found to be a score of ≥3. Additionally, the PEWS score of 3 was closely related to the reference standard in the EOR (Supplementary Material S3). In patients with influenza, there was a Spearman's correlation of 0.454 between the highest score within 24 h observation and discharge. The discharge and highest PEWS scores in the EOR were significantly different (*P* < 0.001).

**Table 3(A):** Cutoff values of PEWS scores among children infected with influenza and unplanned readmission.

Cutoff scores				
Final PEWS upon discharge	Sensitivity	Specificity	LR+	LR−
0	100	0	1	N/A
<1, ≥1	91.8	52.4	1.9	0.2
<2, ≥2	40.8	98.6	29.2	0.6
<3, ≥3	4.1	100	N/A	1.0
<4, ≥4	0	100	N/A	1.0
Highest PEWS on admission				
<1, ≥1	93.9	38.1	1.5	0.2
<2, ≥2	91.8	74.8	3.6	0.1
<3, ≥3	61.2	96.6	18.0	0.4
<4, ≥4	14.3	100	N/A	0.9
<5, ≥5	0.04	100	N/A	1.0

**Table 3(B):** Cutoff values of PEWS scores in readmitted children infected with influenza and adjusted PEWS scores.

Cutoff scores				
Adjusted PEWS on admission	Sensitivity	Specificity	LR+	LR−
<1, ≥1	100	0	1	N/A
<2, ≥2	95.9	37.4	1.5	0.1
<3, ≥3	95.9	72.8	3.5	0.1
<4, ≥4	61.2	95.2	12.8	0.4
<5, ≥5	16.3	99.3	23.3	0.8
Adjusted PEWS upon discharge				
0	100	0	1.0	N/A
<1, ≥1	95.9	41.5	1.6	0.1
<2, ≥2	40.8	5.4	7.6	0.6
<3, ≥3	0.02	100	N/A	1.0
<4, ≥4		100	N/A	1.0

## 4 Discussion

Previous research discussed the use of PEWS systems in identifying clinical deterioration in hospitalized children and emphasized the necessity for larger validation to ensure its reliability among diverse conditions (25). This study extended this application to the emergency department based on constant observations, indicating the adaptability of PEWS systems beyond inpatient units.

The central nervous system and lungs are the organ systems most severely impacted in children who were affected with influenza. The respiratory domain of PEWS includes tachypnea, increased breathing effort, hypoxemia, and an increasing need for inhalation therapy as a result of progressive lower respiratory tract involvement. Simultaneously, the behavioral component of the score reflects influenza-associated hyperpyretic convulsion, or early changes in mental status that show up as irritability, lethargy, or decreased responsiveness. These findings might support the use of PEWS as a structured bedside tool to monitor the two most vulnerable organ systems in pediatric influenza and to facilitate earlier recognition of patients who may benefit from further treatment or care escalation. Provisional validation of a pediatric early warning score for resource-limited settings (PEWS-RL) achieved a specificity of 87.3% for identifying clinical deterioration via a simple score on account of vital signs, mental status, and presence of respiratory distress (26). In our study, a high specificity of up to 96.6% was observed, which aligned with validation work on PEWS adaptations. However, PEWS-RL achieved a higher sensitivity of 96%; our research obtained a lower sensitivity of 61.2%, which may reflect specific cohort factors like influenza-related variability and different outcome definitions (27). In a previous study exploring PEWS in hospitalized pediatric oncology and hematopoietic stem cell transplant patients, the elevated PEWS scores were highly related to unplanned ICU transfer with an AUC of 0.96 (28), as well as PICU mortality (28). It was consistent with our study that the increased risk of unplanned readmission among influenza patients with rising PEWS scores (OR 9.98–10.53), which suggested PEWS may serve as a tool for early recognition of clinical deterioration in high-risk individuals. Context-based risk stratification of PEWS made it possible to identify at-risk patients much earlier than vital sign deviations, and it paved the way for developing next-generation warning systems (29). This detected deteriorated symptoms up to 24 h in advance with a high sensitivity of 0.70, which was also consistent with our results of longer time reaching peak PEWS scores in readmitted patients with influenza. Thus, early trend monitoring among high-risk patients is probably more important than a single threshold of documenting scores.

Among these, 49 children met the inclusion criteria, including unplanned return to EOR within 72 h with similar symptoms and complete PEWS scores. We found a significant association between the highest PEWS score in the first 24 h observation and the final score when discharged for the same clinical symptom within 72 h. PEWS scores were higher for those who were readmitted unplanned than for those who were not. These results suggest that PEWS scores may have the potential to identify influenza-infected children at risk of unplanned readmission. A vital risk index (VRI) of PEWS in an automated and objective method reported AUC at false-positive rates ≤ 10% of 0.064, compared with conventional PEWS of 0.064, *P* = 0.74. The AUC of VRI when recognizing patients in danger of clinical deterioration was 0.76 (95% CI: 0.72–0.80) (30). It showed a comparable AUC performance to the highest PEWS on admission as 0.89 in our study. However, our present study lacked real-time data integration of VRI, which may provide an improvement for further investigation of continuous monitoring data.

Previous studies have used a PEWS cutoff value above 3 to indicate clinical deterioration, and it was stressed that a total score above 4 or a single score of 3 can serve as a valuable warning for CDEs (31). In this study, a cutoff value of 3 for the highest score observation predicted clinical deterioration in severe influenza among children. According to the study results, approximately 17.8% of the children exhibited complications, which underscored the need for careful consideration and potential modification of these criteria to minimize false positives based on a particular situation. Zoham et al. (32) found that patients with elevated PEWS scores had an increased risk of readmission to the PICU than those with lower scores. However, the link between the highest and discharged PEWS scores and the likelihood of readmission among children affected by influenza has rarely been explored. Our study focused on the association between the highest PEWS score during observation and the final score at discharge in the pediatric EOR. Increased PEWS scores could be a valuable tool for screening high-risk influenza-infected children, and cutoff PEWS scores could be an essential factor for discharge decisions or the necessity of unplanned readmission. This study is the first to investigate the association between the highest PEWS score during the first 24 h observation and the final discharge score, which is responsible for unplanned readmission in influenza-affected children in the pediatric emergency department.

A previous study found that patients with influenza frequently encounter significant risk factors requiring inhalation medications, which could be linked to unplanned readmissions (33, 34). The regression model was hypothesized to use inhalation medications consistently to differentiate between the primary and evolving PEWS scores in at-risk patients. This study established a referential PEWS cutoff score capable of distinguishing between low- and high-risk pediatric patients. Although the PEWS cutoff score of 2 exhibited a sensitivity and specificity of 91.8% and 74.8%, respectively, particularly when children were observed during the observation period, this threshold proved insufficient to independently determine the need for unplanned readmission. Using a PEWS cutoff of 2 for readmission decisions can be challenging because of the low PLR of 3.6 and NLR of 0.1. To enhance the statistical power of this study, we adopted a 3:1 case–control ratio. PEWS scores of 3 or greater exhibited an increase in likelihood ratio estimates and were proven to be more accurate values for predictive performance. Thus, the high specificity of 96.6% and PLR up to 18.0 indicated PEWS might be effective in identifying influenza children in the emergency observation department requiring intervention, potentially reducing readmission rates through timely antiviral initiation and discharge plans.

Elevated levels of PCT, ALT, and CK were observed in the unplanned readmission group, consistent with previous literature (35–38). These markers were indicated with bacterial co-infection, hepatic involvement (39, 40), and viral myositis (41, 42) in severe influenza. These findings support the utility of PEWS as a non-invasive tool, which combined with laboratory parameters may provide additional insight into organ-specific dysfunction. In the unplanned readmission group, ALT elevation also exhibited a significant difference from the no readmission group (*P* = 0.001). CK elevation was observed in the unplanned readmission group with influenza, compared with the no readmission group (*P* < 0.001). This also implied the importance of early recognition of elevated PEWS scores, and resolving with specific treatment to prevent worsening progressively.

### 4.1 Limitations

This study had several limitations. Rather than being a multicenter, long-term study, this was a retrospective, single-center, case–control clinical study that lasted for 6 months. The influenza virus mutation may have produced inconsistent results. Second, the influenza patients were children from southwestern China, and a modified version of the PEWS was used in this study. These criteria may vary from hospitals, as hospitals may use a different system especially designed for their patients. Additionally, the PEWS system used in this study included modifications such as additional points for continuous inhalation medication, which might make it more difficult to compare directly with validated versions used in different settings. Although these modifications increased contextual relevance, they may affect the generalizability of our results when applied to organizations that use PEWS without any modifications. Thus, our study results may not be applicable to all institutions, or they may be contrary to the findings of other studies.

### 4.2 Implications for use in the PEWS program

The PEWS can effectively identify clinical deterioration in severe influenza through continuous monitoring and score recording. A PEWS score exceeding 3 and a final PEWS score exceeding 1 may serve as early warnings for unplanned readmission within 3 days. This is particularly significant because a previous study that used the PEWS in the pediatric emergency department did not establish an alarm system to assist in assigning PEWS scores for patient management.

## 5 Conclusions

PEWS may be useful as a supportive tool for evaluating discharge safety in children with influenza, according to this single-center case–control study. However, considering the retrospective design, single-center setting, and small sample size, these results should be interpreted cautiously. To verify the generalizability and clinical applicability of PEWS in this context, additional validation through multicenter, prospective studies is suggested. Notably, an observation PEWS score exceeding 3 and a discharge score surpassing one serve as alert thresholds for electronic documentation, signaling cases of severe influenza with a potential for clinical deterioration.

## Data Availability

The original contributions presented in the study are included in the article/Supplementary Material, further inquiries can be directed to the corresponding author.
