# Supplementary material for: Pediatric early warning score and unplanned readmission with influenza at emergency observation room
Source: Front Pediatr. 2026 Jan 16;13:1674746. doi: 10.3389/fped.2025.1674746 (PMC12855074; doi:10.3389/fped.2025.1674746)
Supplement: Supplementary file 1 [file Supplementaryfile1.docx]

**Supplementary material 1 The pediatric early warning score revised in this research**

|  | **0** | **1** | **2** | **3** |
| --- | --- | --- | --- | --- |
| Behavior | Playing/  appropriate | Sleeping | Irritable | Lethargic/confused or reduced response to pain |
| Cardiovascular | Pink or capillary refill 1-2 seconds | Pale or capillary refill 3 seconds | Gray or capillary refill 4 seconds or tachycardia of ≥20 bpm above normal rate | Gray and mottled or capillary refill ≥ 5 seconds or tachycardia≥30 bpm above normal rate or bradycardia |
| Respiratory | Within normal parameters, no retractions or tracheal tug | Respiratory rate ≥10 breaths/min above normal parameters, using accessory musles or 30%+FiO_2_ or 3+L/min | Respiratory rate ≥20 breaths/min above normal parameters, retractions, tracheal tug, or 40%+FiO2 or 6+L/min | RR 5 breaths/min below normal rate with retractions and/or grunting, or 50%+ FiO2 or 8+L/min |

doi:10.1371/journal.pone.0072534.t001

**Supplymentary material 2 Normal range of vital signs with different age categories**

| **Categories** | Age | Heart rate | **Respiratory rate** |
| --- | --- | --- | --- |
|  |  | (beats / minute) at rest | (breaths / minute) at rest |
| Child | 2~3years | 70-110 | 25-30 |
| Preschooler | 4~6years | 70-110 | 21-23 |
| School-age child | 7~12years | 70-110 | 19-21 |
| Adolescent | 13~14years | 55-90 | 16-18 |

doi:10.1371/journal.pone.0072534.t002

**Supplymentary material 3 Distribution of severe influenza deterioration by the reference standard and estimated by PEWS, among the children recruited**

| **Actual influenza deterioration** | n | % |
| --- | --- | --- |
| By the reference standard | 17.8 | 17.8 |
| By the PEWS≥3 | 20 | 20.0 |
| By the PEWS≥4 | 14 | 14.0 |
